# Critically Ill Patients with Visceral *Nocardia* Infection, France and Belgium, 2004–2023

**DOI:** 10.3201/eid3002.231440

**Published:** 2024-02

**Authors:** Lucas Khellaf, Virginie Lemiale, Maxens Decavèle, Marc Pineton de Chambrun, Alexandra Beurton, Toufik Kamel, Anabelle Stoclin, Djamel Mokart, Fabrice Bruneel, Clara Vigneron, Achille Kouatchet, Benoît Henry, Jean-Pierre Quenot, Grégoire Jolly, Nahema Issa, Matthieu Bellal, Julien Poissy, Claire Pichereau, Julien Schmidt, Nathalie Layios, Maxime Gaillet, Elie Azoulay, Adrien Joseph

**Affiliations:** Saint-Louis Teaching Hospital, Public Assistance Hospitals of Paris (APHP), Paris, France (L. Khellaf, V. Lemiale, E. Azoulay, A. Joseph);; Pitié-Salpétière Teaching Hospital, APHP, Paris (M. Decavèle, M. Pineton de Chambrun);; Hôpital Tenon, Groupe Hospitalo-Universitaire Sorbonne University, APHP, Paris (A. Beurton);; Centre Hospitalier Régional d’Orléans, Orléans, France (T. Kamel); Institut Gustave Roussy, Villejuif, France (A. Stoclin);; Institut Paoli Calmettes, Marseille, France (D. Mokart);; Centre Hospitalier de Versailles, Hôpital André Mignot, Le Chesnay, France (F. Bruneel);; Cochin Teaching Hospital, APHP, Paris (C. Vigneron);; Centre Hospitalier Universitaire d’Angers, Angers, France (A. Kouatchet);; Hôpital Bicêtre, APHP, France (B. Henry);; Dijon Bourgogne University Hospital, Dijon, France (J.-P. Quenot);; Rouen University Hospital, Rouen, France (G. Jolly);; Saint-André Hospital, Bordeaux, France (N. Issa);; University Hospital of Caen, Caen, France (M. Bellal);; Hôpitaux Universitaires de Strasbourg, Strasbourg, France (J. Poissy);; Centre Hospitalier Intercommunal de Poissy Saint Germain, Poissy, France (C. Pichereau);; Avicennes Hospital, APHP, Ile de France, France (J. Schmidt);; University Hospital of Liege, Liege, Belgium (N. Layios);; Lyon University Hospital, Lyon, France (M. Gaillet)

**Keywords:** *Nocardia*, immunocompromised, intensive care, bacteria, France, Belgium

## Abstract

We studied 50 patients with invasive nocardiosis treated during 2004–2023 in intensive care centers in France and Belgium. Most (65%) died in the intensive care unit or in the year after admission. *Nocardia* infections should be included in the differential diagnoses for patients in the intensive care setting.

*Nocardia* is a ubiquitous, filamentous, gram-positive bacillus present in soil and decaying plants (*1*), affecting immunocompromised patients by way of inhalation, with a risk of secondary dissemination. Invasive *Nocardia* infections are mainly observed in patients who have undergone organ transplantation (incidence 0.2%) and hematopoietic stem cell transplantation (incidence 1.7%). Infections also occur in persons with primary immunodeficiency, solid cancer, or autoimmune disease. Other previously identified risk factors include use of long-term steroids and calcineurin inhibitors (*2*–*4*). Pulmonary involvement constitutes the most common manifestation of *Nocardia* infection, which can potentially lead to secondary dissemination, particularly in immunocompromised populations; the central nervous system is a common site, and many cases involving asymptomatic manifestations (*5*).

Blood cultures are positive in 10%–20% of cases involving *Nocardia* infection, and lung PCR can indicate colonization, requiring such tests as bronchoalveolar lavage and abscess needle aspiration. *Nocardia* species are typically resistant to common antibiotics, which contribute to the complexity of diagnosing and managing disseminated infections (*6*,*7*). The mortality rate associated with *Nocardia* infection is substantial; 16%–40% of patients die within the first year of diagnosis, and outcomes depend largely on the underlying disease (*6*–*8*). We explored the risk factors, characteristics, and prognosis of patients with invasive nocardiosis in the context of the intensive care setting.

## The Study

We conducted a retrospective, multicenter study of patients with invasive nocardiosis admitted to 22 intensive care units (ICUs) from the Groupe de Recherche Respiratoire en Réanimation Onco-Hématologique (Grrr-OH) during 2004–2023 in France and Belgium. We established inclusion criteria as unplanned ICU medical admission, age >18 years, and a documented invasive nocardiosis diagnosis (before or during ICU stay). We excluded cases of suspected nocardiosis without microbiological documentation or those with a lack of medical chart data.

Documented nocardiosis was determined by a positive culture for *Nocardia* species or a *Nocardia* PCR-based assay coupled with organ involvement. Disseminated nocardiosis was characterized by the infection affecting >2 noncontiguous sites; bacteremia constituted dissemination if 1 organ was involved. Organ failures were identified based on the Sepsis-related Organ Failure Assessment score.

We performed a comparison between patients admitted to the ICU for *Nocardia* infection and patients enrolled in the HIGH multicenter clinical trial (*9*), which included immunocompromised patients admitted to the ICU for acute respiratory failure and compared the effect of high-flow nasal oxygen versus standard oxygen on 28-day mortality. We excluded diagnoses of *Pneumocystis* infection, acute pulmonary edema, and specific lung lesions, which we assumed could be easily distinguished from nocardiosis.

We present continuous data as median (interquartile range) and categorical data as numbers and percentages. We compared characteristics between our cohort and data from the HIGH clinical trial by using a Wilcoxon rank-sum test (continuous variables) or Fisher exact test (categorical variables). We used only variables that were statistically significant (p<0.05) in univariate analysis in multivariate analysis and conducted an assessment of collinearity. We performed 2-sided statistical analyses by using R statistical software version 2023.03.0+386 (The R Foundation for Statistical Computing, https://www.r-project.org).

In total, we studied 50 patients with invasive nocardiosis who were admitted to the ICU. The median age was 59 (47–67) years; 39 (78%) were men, and 11 (22%) were women. We took into account such details as patient demographics, concurrent diseases, and immunosuppressive therapies (Table 1). Almost all patients (46 [92%]) were immunocompromised; the primary causes were solid organ transplantation (18 [36%]), systemic autoimmune diseases (12 [24%]), and hematologic malignancies (10 [20%]). Steroid therapy was administered to most patients (34 [68%]). Low-dose trimethoprim/sulfamethoxazole prophylaxis was given to 12 (24%) patients.

**Table 1:** Baseline characteristics of patients in study of critically ill patients with visceral *Nocardia* infection, France and Belgium, 2004–2023*

Baseline characteristics	Nocardiosis cases, n = 50
Age, y, median (IQR)	59 (47–67)
Sex	
M	39 (78)
F	11 (22)
Charlson score, median (IQR)	4 (3–7)
Cardiovascular risk factors, median (IQR)	2 (1–3)
Immunosuppression	46 (92)
Corticosteroids at admission	34 (68)
5–10 mg/d	5 (10)
>10 mg/d	29 (58)
Tacrolimus treatment	16 (32)
Mycophenolate mofetil treatment	12 (24)
Other conventional immunosuppressive drugs†	5 (10)
Organ transplantation	18 (36)
Kidney	12 (24)
Heart	4 (8)
Liver	1 (2)
Lung	1 (2)
Systemic autoimmune disease‡	12 (24)
Hematologic malignancies	10 20)
Aggressive B cell lymphoma	6 (12)
Acute lymphoid leukemia	3 (6)
Acute myeloid leukemia	1 (2)
No. lymphocytes/mm^3^, median (IQR)	552 (287–1,210)
Gamma globulin, g/L, median (IQR)	6 (4–10)
Trimethoprim/sulfamethoxazole prophylaxis	12 (24)

*Values are no. (%) except as indicated. IQR, interquartile range. †Azathioprine, n = 4 (8%); methotrexate, n = 1 (2%). ‡Connective tissue disease, n = 2; glomerulonephritis, n = 1; periarteritis nodos, n = 1; bullous pemphigoid, n = 1; Evans syndrome, n = 1; IgA vasculitis, n = 1; type 1 diabetes, n = 1; myasthenia gravis, n = 1; sarcoidosis, n = 1; chronic inflammatory demyelinating polyradiculoneuropathy, n = 1; inflammatory bowel disease, n = 1.

We noted disseminated infection in almost half of the patients (48%); the most frequently involved organs were lungs (98%), central nervous system (47%), and skin (20%) (Table 2). At admission to intensive care, 33 (66%) patients had acute respiratory distress and 19 (38%) experienced coma (defined by a Glasgow Coma Scale score ≤8) or septic shock. Overall, 45 (90%) patients exhibited >1 organ failure; the most common were respiratory failure (33 [66%]) and multiorgan dysfunction (25 [50%]). Computed tomography scans revealed alveolar consolidations in 43 (86%) patients and cavitated nodules in 26 (52%) patients. Magnetic resonance imaging of the brain in 23 (46%) patients revealed multiple lesions in 14 (61%) patients and brain herniation in 6 (26%) patients.

**Table 2:** Patient clinical and radiologic findings from the intensive care unit in study of critically ill patients with visceral *Nocardia* infection, France and Belgium, 2004–2023*

Findings	Nocardiosis cases, n = 50
Clinical features	
Chronic cough†	36 (72)
No. previous antibacterial therapy lines	2 (0–3)
Fever	29 (58)
Co-infection	22 (44)
Fungal‡	11 (22)
Bacterial§	8 (16)
Viral¶	5 (10)
Lung involvement	49 (98)
Oxygen therapy at admission	32 (64)
Oxygen flow, L/min, median (IQR)	8 (4–15)
Respiratory rate, L/min, median (IQR)	30 (25–36)
Hemoptysis	8 (16)
Neurologic involvement	24 (48)
Confusion	21 (42)
Coma	16 (32)
Motor deficit	13 (26)
Cranial nerve lesions	10 (20)
Meningitis	8 (16)
Epilepsy	6 (12)
Glasgow score, median (IQR)	13 (12–14)
Skin/muscle abscess	10 (20)
Disseminated infection	24 (48)
Organ failures	45 (90)
Multiorgan	25 (50)
Respiratory	33 (66)
Including acute respiratory distress syndrome	3 (8)
Acute kidney injury	11 (22)
Hemodynamic	17 (34)
Neurologic	19 (38)
Hepatic	4 (8)
Sequential organ failure assessment score, median (IQR)	5 (3–7)
Imaging findings	
Computed tomography scan	
Lung consolidation	43 (86)
Lung nodules with cavitation	26 (52)
Pleural effusion	15 (30)
Interstitial syndrome	8 (16)
Alveolar hemorrhage	6 (12)
Lung lobes involved	
1 lobe	16 (32)
Multilobe	16 (32)
Bilateral	18 (36)
Brain magnetic resonance imaging, n = 23	
Single lesion	9 (39)
Multiple lesions	14 (61)
>10 mm	17 (74)
<10 mm	6 (26)
Brain herniation	6 (26)
Ventriculitis	2 (9)
Diagnostics methods	
Bronchoalveolar lavage analysis	42 (84)
Diagnostic yield, n = 42	25 (60)
Computed tomography–scan targeted biopsy	18 (36)
Blood culture positivity	7 (14)
* Nocardia* PCR-based assay positivity	26 (52)
* Nocardia* culture positivity	24 (48)
Diagnosis made in intensive care unit	23 (46)

*Values are no. (%) except as indicated. IQR, interquartile range. †Chronic cough is defined as a cough persisting for >8 weeks. ‡Fungal infections (n = 11) comprised 8 invasive *Aspergillus* sp. infections, 2 *Pneumocystis jirovecii* infections, and 1 case of cutaneous candidosis. §Comprised 7 gram-negative bacillus co-infections and 1 methicillin-resistant *Staphylococcus aureus* co-infection. ¶Virus infections (n = 5) comprised 3 influenza infections (including 1 H1N1 co-infection) and 2 respiratory syncytial virus infections.

Most (62%) patients received dual therapy or triple therapy, including aminoglycosides (10 [20%]), most commonly trimethoprim/sulfamethoxazole (80%) and carbapenem (51%) (Appendix Table 1). Upon admission to the intensive care unit, 32 patients (63%) required oxygen support, and 19 (38%) required mechanical ventilation. The ICU mortality rate was 22%, and the all-cause mortality rate at 1 year was 44%. In multivariable analysis, factors significantly associated with 1-year mortality included vasopressor use, fungal coinfection, and neurologic involvement (Appendix Table 2).

We compared cases of *Nocardia* infection against cases of other immunocompromised pneumonia in patients admitted to the ICU (*2*,*11*) (Figure; Appendix Table 3). Patients with *Nocardia* infection were younger and had a higher prevalence of autoimmune diseases and solid organ transplants. Lung consolidation (86% vs. 27%; p = 0.001) and cavitated nodules (52% vs. 1%; p = 0.001) were significantly more frequent. Upon admission to the ICU, patients with nocardiosis were rated as more severe on the Sepsis-related Organ Failure Assessment and Glasgow Coma Scale compared with patients with other immunocompromised pneumonia, but there was no significant difference in ICU mortality (22% vs. 32%; p = 0.184).

**Figure:**
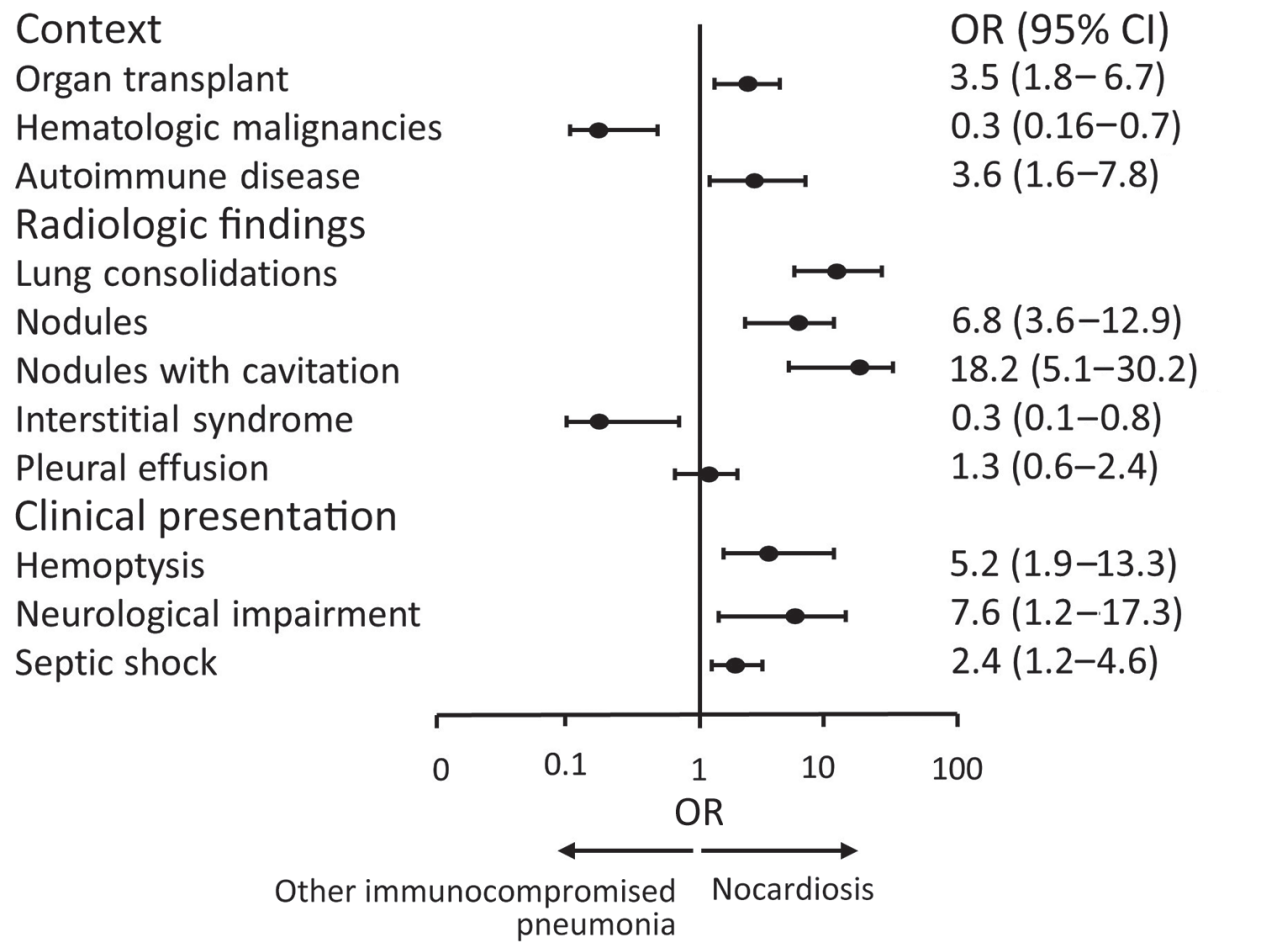
Comparison between nocardiosis and other immunocompromised pneumonia in a study of patients admitted to intensive care units in France and Belgium during 2004–2023. Other immunocompromised pneumonia data extracted from the HIGH clinical trial (*9*). OR, odds ratio.

## Conclusions

Based on the findings for our study population, critically ill patients with nocardiosis exhibit frequent and severe pulmonary and neurologic involvement; 44% of patients die (22 of 50) and 14% (7 of 50) experience disability at the 1-year mark. Several cohorts have documented *Nocardia* infections within diverse immunocompromised populations, reporting mortality rates of 16%–40% (*3*,*4*). In our analysis, we conducted a comparative assessment with other pneumonia cases in immunocompromised patients (*9*) to elucidate situations warranting consideration of *Nocardia* infection. Cellular immunosuppression appears to be necessary for the development of a severe *Nocardia* infection, which is consistent with previous studies (*4*,*6*,*10*), particularly among organ transplant recipients, patients with systemic autoimmune diseases, and those with hematologic malignancies. Co-infections, particularly fungal ones, were reported as an independent prognostic factor for mortality in this population (*11*) and could partially explain this initial severity. Such findings highlight the burden of immunosuppression and the need for vigilance in assessing concurrent infections in this population. Two recent studies suggest that trimethoprim/sulfamethoxazole could be protective against *Nocardia* infections (*11*,*12*). Because invasive *Nocardia* infections are rare, results of our study may lack statistical power, and significant prognostic or distinctive factors might have gone unnoticed. However, we believe the inclusion of patients from 22 ICUs, with few cases missing data, provides a relevant overview of nocardiosis in critically ill patients.

In summary, in this study of critically ill patients with nocardiosis, we observed high mortality rates, posing a diagnostic challenge for critical care practitioners. Our findings emphasize the need for a heightened level of vigilance in monitoring patients for *Nocardia* infection in the intensive care setting, especially among immunocompromised patients who exhibit pulmonary nodules and neurologic involvement.

Appendix Additional information regarding critically ill patients with visceral *Nocardia* infection, France and Belgium, 2004–2023.
