# Supplementary material for: Critically Ill Patients with Visceral Nocardia Infection, France and Belgium, 2004–2023
Source: Emerg Infect Dis. 2024 Feb;30(2):345–9. doi: 10.3201/eid3002.231440 (PMC10826782; doi:10.3201/eid3002.231440)
Supplement: Appendix — Additional information regarding critically ill patients with visceral Nocardia infection, France and Belgium, 2004–2023. [file 23-1440-Techapp-s1.pdf]

Article DOI: http://doi.org/10.3201/eid3002.231440

EID cannot ensure accessibility for supplementary materials supplied by authors. Readers who have difficulty accessing supplementary content should contact the authors for assistance.

Critically Ill Patients with Visceral *Nocardia* Infection, France and Belgium, 2004–2023

Appendix

Appendix Table 1. Management and outcomes in study of critically ill patients with visceral *Nocardia* infection, France & Belgium, 2004–2023*

Therapeutic management	Nocardiosis cases, n = 50
Antibacterial therapy	
Bi-therapy	31 (62)
Tri-therapy including 1 aminoglycoside	10 (20)
Trimethoprim/sulfamethoxazole	40 (80)
Carbapenem	26 (51)
Amikacin	17 (33)
Linezolid	6 (12)
Organ support	
Mechanical ventilation	19 (38)
Duration of mechanical ventilation, median d (IQR)	8 (4–20)
Vasopressors	17 (34)
Dialysis	8 (16)
ECMO	3 (6)
Outcomes	
ICU mortality	11 (22)
Hospital mortality	16 (32)
1-y mortality	22 (44)
Performance status ≥3 at 1 y	7 (14)
ICU length of stay, median d (IQR)	8 (3–23)

*Values are no. (%) except as indicated. ECMO, extracorporeal membrane oxygenation; ICU, intensive care unit; IQR, interquartile range.

Appendix Table 2. Factors significantly associated with 1-year all-cause mortality in study of critically ill patients with visceral *Nocardia* infection, France, 2004–2023*

	Univariate analysis		Multivariate analysis	
Characteristics	OR (95% CI)	p value	OR (95% CI)	p value
Vasopressors use	10.2 (2.7–45.1)	0.001	40.8 (5.7–898.0)	0.002
Neurologic involvement	6.2 (1.8–23.5)	0.004	17.9 (2.7–365.0)	0.012
Fungal co-infection	8.3 (1.8–60.5)	0.014	21.5 (2.2–560.7)	0.020

*OR, odds ratio.

Appendix Table 3. Comparison between the HIGH cohort and *Nocardia* infections in study of critically ill patients with visceral *Nocardia* infection, France, 2004–2023*

Characteristics	HIGH cohort,† n = 342	*Nocardia* cohort, n = 50	p value
Age, mean (IQR)	63 (56–71)	59 (47–67)	0.015
Sex			
M	223 (65)	39 (78)	0.102
F	119 (35)	11 (22)	0.165
Hypertension	132 (38)	36 (72)	0.001
Ischemic heart disease	34 (10)	10 (20)	0.062
Active smokers	57 (17)	25 (50)	0.001
Diabetes	75 (22	14 (28)	0.438
Immunosuppression	339 (99)	46 (92)	0.131
Systemic autoimmune disease	24 (7)	11 (22)	0.001
Hematologic malignancies	124 (36)	10 (20)	0.001
Acute myeloid leukemia	49 (14)	1 (2)	NA
Acute lymphoid leukemia	9 (3)	3 (6)	NA
Aggressive B cell lymphoma	40 (12)	6 (12)	NA
Myeloma	26 (8)	0 (0)	NA
Allogenic graft	31 (9)	2 (4)	0.351
Autologous graft	26 (8)	3 (6)	0.908
Solid tumor	120 (35)	3 (6)	0.001
Solid organ transplantation	46 (14)	18 (36)	0.001
Kidney	29 (9)	12 (24)	NA
Lung	3 (1)	1 (2)	NA
Heart	6 (2)	4 (8)	NA
Liver	6 (2)	1 (2)	NA
Immunosuppressant	126 (37)	46 (92)	0.001
Steroids	82 (24)	34 (68)	0.001
Neutropenia	103 (30)	3 (6)	0.005
Bactrim prophylaxis	59 (17)	12 (24)	0.453
Charlson score at admission, mean (IQR)	5 (3–7)	4 (3–7)	0.330
Computed tomography scan			
Lung consolidation	93 (27)	43 (86)	0.001
Lung nodules with cavitation	15 (4)	26 (52)	0.001
Pleural effusion	86 (25)	15 (30)	0.576
Interstitial syndrome	18 (5)	8 (16)	0.773
Alveolar hemorrhage	21 (6)	6 (12)	0.134
Lung lobes involved			
1 lobe	52 (15)	16 (32)	0.065
Multilobes	21 (6)	16 (32)	0.001
Bilateral	89 (26)	18 (36)	0.452
Clinical data at admission			
SOFA score (IQR)	3 (1–5)	5 (3–7)	0.001
Glasgow score (IQR)	15 (14–15)	13 (12–14)	0.003
Oxygen flow, L/min (IQR)	10 (6–15)	8 (4–15)	0.423
Respiratory rate/min (IQR)	33 (28–39)	30 (25–36)	0.342
Hemoptysis	12 (4)	8 (16)	0.036
Productive cough	175 (51)	36 (72)	0.045
Acute respiratory failure	294 (86)	18 (36)	0.001
Coma	15 (4)	16 (32)	0.001
Septic shock	56 (16)	16 (32)	0.014
Acute kidney failure	46 (14)	10 (20)	0.316
ICU organ support			
Mechanical ventilation	140 (41)	19 (38)	0.785
Duration of mechanical ventilation, d (IQR)	4 (0–7)	8 (4–20)	0.093
Vasopressors	159 (48)	17 (34)	0.835
Dialysis	26 (8)	8 (16)	0.009
ECMO	3 (1)	3 (6)	0.038
Outcomes			
ICU length of stay, d (IQR)	7 (4–13)	8 (3–23)	0.652
ICU mortality	111 (32)	11 (22)	0.184
Hospital mortality	20 (41)	16 (32)	0.110

*Values are no. (%) except as indicated. ECMO, extra corporeal membrane oxygenation; ICU, intensive care unit; IQR, interquartile range; NA, not applicable; SOFA, sequential organ failure assessment.
†Comparisons were made between patients admitted to the ICU for *Nocardia* infection in this study and patients enrolled in the HIGH multicenter clinical trial (*9*, main text).
